# Plasma-Free Metanephrine and Normetanephrine Quantification for Clinical Applications Validated by Combining Solid-Phase Extraction and HPLC-MS/MS

**DOI:** 10.3390/molecules30193847

**Published:** 2025-09-23

**Authors:** Hyebin Choi, Jisook Yim, Jiwon Yun, Jong Kwon Lee, Keun Ju Kim, Minjeong Nam, Myung Hyun Nam, Yunjung Cho, Seung Gyu Yun

**Affiliations:** Department of Laboratory Medicine, Korea University Anam Hospital, Seoul 02841, Republic of Korea; chb1996@naver.com (H.C.); newsroomw@gmail.com (J.Y.); drjwyun@kumc.or.kr (J.Y.); cr99ljk@gmail.com (J.K.L.); themicrobialworld@gmail.com (K.J.K.); mjnam0906@gmail.com (M.N.); yuret@korea.ac.kr (M.H.N.); eqcho1ku@korea.ac.kr (Y.C.)

**Keywords:** metanephrine, normetanephrine, LC-MS/MS, solid-phase extraction, pheochromocytoma, bioanalytical method validation, plasma analysis

## Abstract

Plasma-free metanephrines are the most sensitive and specific biochemical markers for diagnosing catecholamine-secreting tumors, such as pheochromocytoma and paraganglioma. In this study, we developed and validated a liquid chromatography–tandem mass spectrometry method for quantifying metanephrine and normetanephrine in human plasma, using solid-phase extraction with a weak cation-exchange mechanism. Validation was performed according to the FDA Bioanalytical Method Validation Guidance and CLSI guideline C62-A. The method showed excellent linearity over concentration ranges of 0.11–13.92 nmol/L for metanephrine and 0.14–26.43 nmol/L for normetanephrine, with correlation coefficients exceeding 0.999. The accuracy, precision, and lower limit of quantification met the acceptance criteria of the study. Matrix effect evaluation revealed a process efficiency of 121% for metanephrine at the lowest concentration, slightly exceeding the acceptable range of 100 ± 15%. This was likely because of matrix-induced ion enhancement or variability in extraction efficiency. However, all other tested concentrations were within the acceptable limits. Overall, this method demonstrated high sensitivity, specificity, and reproducibility, making it suitable for routine clinical applications. Minor deviations at low concentrations do not compromise reliability; however, future optimizations, such as matrix-matched calibration, may further improve performance.

## 1. Introduction

Metanephrine and normetanephrine are O-methylated metabolites of catecholamines secreted by the adrenal medulla [1,2]. Metanephrines are synthesized from L-tyrosine [3], which is converted into dihydroxyphenylalanine (DOPA) by tyrosine hydroxylase before being converted to dopamine by DOPA decarboxylase [4]. Dopamine is then converted to norepinephrine and epinephrine, which are finally converted to metanephrine by catechol-*O*-methyltransferase.

Some adrenal gland tumors produce excess catecholamines, which are broken down to produce metanephrines. The most common adrenal gland tumors are pheochromocytomas and neuroblastomas. Pheochromocytomas are paragangliomas that arise within the adrenal gland [5], whereas neuroblastomas arise from neuroblasts and most often occur in the adrenal glands during childhood [6]. 

Pheochromocytomas are primarily diagnosed through biochemical screening. Plasma-free and urinary metanephrine levels are measured first. Compared with metanephrines, epinephrine alone is a less sensitive screening marker [7,8]. Similarly, urinary metanephrine analysis has a lower sensitivity and requires a 24-h urine collection [9,10]. Therefore, the measurement of plasma-free metanephrine levels is preferred for diagnosing pheochromocytomas [11,12]. If plasma levels are less than three times the upper limit of normal, a clonidine suppression test is considered [13]. Levels exceeding three times the upper limit strongly suggest a tumor; in this case, tumor characterization is performed using radiological imaging, such as computed tomography and magnetic resonance imaging [14].

Multiple LC–MS/MS workflows have been reported for plasma-free metanephrines, with differences primarily in sample preparation and chromatographic mode [15,16]. Reported sample-cleanup options include protein precipitation, polymeric reversed-phase (RP) SPE, and weak cation-exchange (WCX) SPE. WCX exploits the basicity of metanephrines, so that the protonated analytes are retained under acidic load steps, and acidified organic solvent affords efficient elution. Chromatographic separation has employed RP columns, which often provide limited retention for these polar analytes, and HILIC phases, which provide improved retention and peak shape under high-organic initial conditions. Some methods also use online SPE and, in selected protocols, derivatization in order to extend sensitivity [17,18]. 

Metanephrines are present at low concentrations in plasma; however, they were concentrated in plasma samples using solid-phase extraction (SPE) [19]. SPE was used to purify and concentrate the analytes based on their physicochemical properties. Because of their basic nature (pKa ≈ 9–9.3), metanephrines are protonated under acidic conditions, which enables their efficient retention on WCX sorbents, while subsequent elution with acidified organic solvent allows effective recovery. Therefore, we evaluated WCX against alternative sorbents and optimized the loading, wash, and elution conditions to maximize recovery while minimizing matrix effects. Subsequently, liquid chromatography and tandem mass spectrometry (LC-MS/MS) were performed to detect metanephrines [20]. LC-MS/MS offers greater sensitivity, specificity, and speed than conventional methods, such as electrochemical and fluorometric detection [21,22,23]. Here, we validated a method for diagnosing pheochromocytomas by detecting metanephrines in plasma using SPE and LC-MS/MS.

## 2. Results

### 2.1. Accuracy

**Figure 1:**
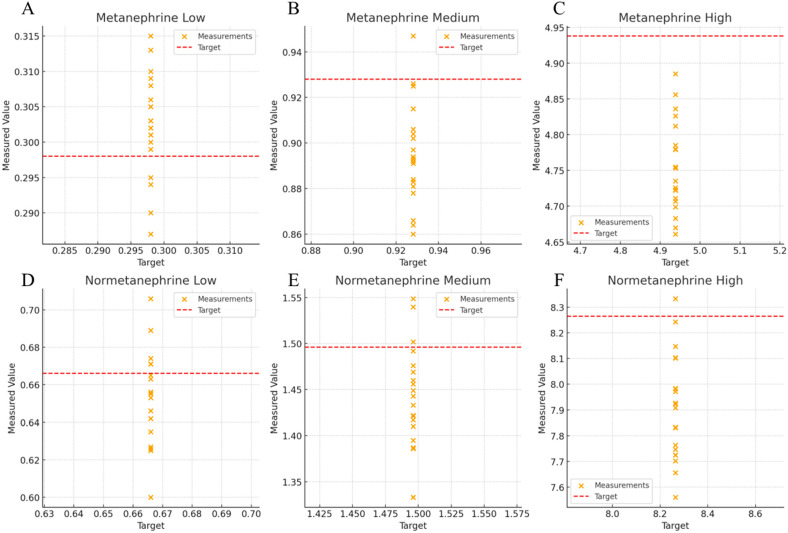
Scatter plot of Metanephrine and Normetanephrine across low, medium, and high concentrations. (**A**) Low concentration, metanephrine; (**B**) Medium concentration, metanephrine; (**C**) High concentration, metanephrine; (**D**) Low concentration, normetanephrine; (**E**) Medium concentration, normetanephrine; (**F**) High concentration, normetanephrine.

The accuracies of metanephrine and normetanephrine were 96.3–101.5% and 95.7–98.1%, respectively (Table 1, Figure 1). Accuracy was evaluated at three concentration levels using the FDA Bioanalytical Method Validation Guidance (±15%; ±20% at lower limit of quantification [LLOQ]) and CLSI C62-A criteria.

### 2.2. Precision

Precision was evaluated using within-run and between-run tests. In the within-run test, identical samples were analyzed for five days. The accuracies were 96.5–99.8%, while the coefficients of variation (CVs) were 1.4–4.2%, satisfying the acceptance criteria. In the between-run test, identical samples were analyzed twice daily, with a time gap of at least 4 h between tests, for 5 d. The accuracies were 93.1–100.7%, and the CVs were 1.7–7.0%, which also satisfied the acceptance criteria (Table 2).

### 2.3. Linearity

**Figure 2:**
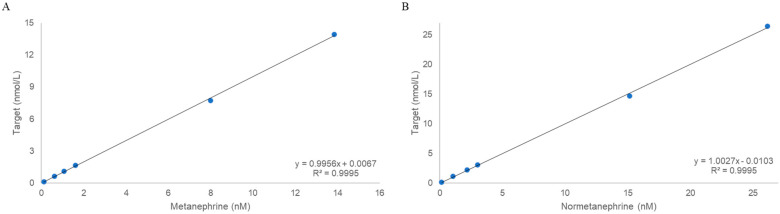
Validation of linearity: (**A**) Metanephrine. (**B**) Normetanephrine.

The concentration ranges used to validate linearity were 0.11–13.92 nmol/L for metanephrine and 0.14–26.43 nmol/L for normetanephrine. The back-calculated accuracies for all calibration levels were within ±15% (±20% at the LLOQ), and the regression yielded R^2^ = 0.9995 (Figure 2). 

### 2.4. Carryover

**Figure 3:**
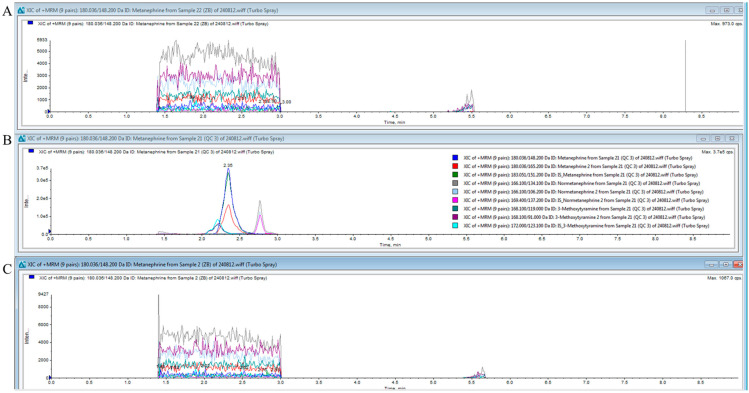
Chromatograms of blank samples before and after analysis. (**A**) shows the chromatogram of the blank before analysis, (**B**) shows the chromatogram of the high-concentration control, and (**C**) shows the chromatogram of the blank after analysis.

An F-test was conducted prior to conducting the *t*-test. The *p*-values of the F-tests were < 0.5, demonstrating that the low 1 and 3 groups had equal variances. Homoscedastic *t*-tests were performed. The *p*-values of the *t*-tests were all <0.05. No statistically significant differences were observed between the two groups (Table 3). Figure 3 shows the chromatograms of the blank samples before and after the analysis. Figure 3A shows the chromatogram of the blank sample before analysis, Figure 3B shows the chromatogram of the high-concentration control, and Figure 3C shows the chromatogram of the blank sample after the analysis. The chromatogram of the blank after analysis is comparable to that before analysis, indicating that no carryover occurred.

### 2.5. Lower Limit of Quantification

The LLOQ was determined by analyzing diluted control samples (Table 4). The expected concentrations were 0.061–0.307 nmol/L for metanephrine and 0.108–0.541 nmol/L for normetanephrines. The lowest concentration that satisfied an accuracy within 100 ± 15% and CV% of <20% was determined. The LLOQ was determined to be 0.123 and 0.432 nmol/L for metanephrine and normetanephrine, respectively. 

### 2.6. Ion Suppression

**Figure 4:**
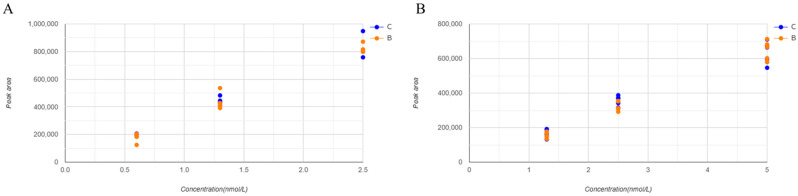
Scatter plot illustrating matrix effect on metanephrines. Each concentration was tested five times. (**A**) shows metanephrine and (**B**) shows normetanephrine.

The matrix effect, recovery, and process efficiency were verified by comparing samples spiked with the drug and internal standards (metanephrine-d_3_ and normetanephrine-d_3_, IS) before (Sample A) and after (Sample C) SPE, and the drug and IS spiked with 90% acetonitrile (Sample B) were compared. The results are presented in Table 5. The peak areas of the samples were normalized to that of the IS. The normalized results are listed in Table 5 and shown in Figure 4. All evaluations were within the acceptable range of 100 ± 15%. The IS-normalized recovery rates were 86–112% and 98–112% for the IS-normalized matrix effect and 92–121% for the IS-normalized process efficiency at all metanephrine concentrations, respectively. The lowest metanephrine concentration was 15%.

### 2.7. Inter-Laboratory Comparison

**Figure 5:**
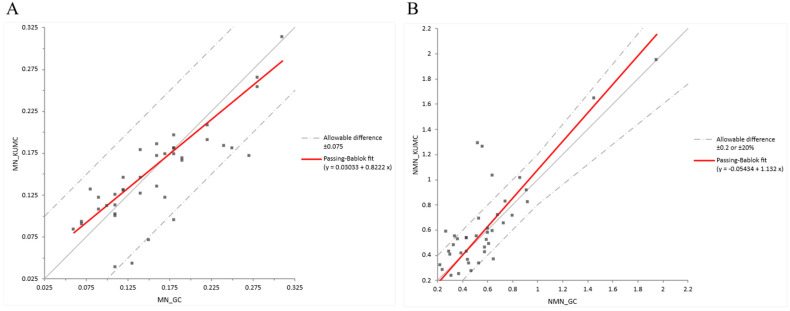
Interlaboratory comparison of metanephrines using Passing–Bablok regression. (**A**) shows metanephrine and (**B**) shows normetanephrines. The grey solid line represents the line of identity (y = x), indicating perfect agreement between the two methods.

Inter-laboratory comparison demonstrated good overall agreement between our laboratory and the Green Cross Corporation. For metanephrine, Passing–Bablok regression analysis yielded a slope of 0.822 (intercept = 0.030), suggesting that our measurements tended to be lower than those from Green Cross (Figure 5A). For normetanephrine, the slope was 1.132 (intercept = –0.054), indicating a proportional positive bias with slightly higher values obtained in our laboratory (Figure 5B). Importantly, most of the results fell within the allowable difference (±0.075 nmol/L for metanephrines and ±0.2 nmol/L or ±20% for normetanephrines), confirming that the observed bias remained within acceptable analytical limits.

## 3. Discussion

Free metanephrines in plasma are recognized as sensitive and specific biochemical markers for screening catecholamine-secreting tumors, particularly pheochromocytomas and paragangliomas, outperforming urinary epinephrine, norepinephrine, and vanillylmandelic acid in terms of diagnostic accuracy. Consequently, the Endocrine Society guidelines recommend plasma-free metanephrines or urinary fractionated metanephrines measured using LC–MS/MS as the first-line screening test, provided that critical pre-analytical factors, including supine collection, EDTA anticoagulation, prompt cooling, and strict drug exclusion, are controlled [24]. In this context, our SPE–LC–MS/MS method was designed to exceed the clinical performance expectations. Validation data confirmed that it achieved sub-nanomolar limits of quantification and excellent linearity, accuracy, and precision across clinically relevant ranges. 

Our fit-for-purpose approach emphasizes robustness and operational simplicity rather than exhaustive multifactor optimization. We based our key choices on established clinical LC-MS/MS practices for basic analytes and then confirmed them through validation performed at our site under our laboratory constraints (instrument platform, throughput, and sample volume). The achieved performance, particularly the stability of retention and acceptability of matrix effects, supports the suitability for routine clinical use, although the study did not aim to reach the very low LLOQs reported for more complex workflows.

The sub-nanomolar limits of quantification achieved using our method (0.123 nmol/L for metanephrine and 0.432 nmol/L for normetanephrine) are comparable to those reported in studies detailing similar LC–MS/MS applications [25,26,27]. The limits of quantification determined by validation were 0.123 nmol/L for metanephrine and 0.432 nmol/L for normetanephrine, defined as the lowest concentrations meeting 100 ± 15% accuracy and CV < 20% (Table 4). These sensitivity levels exceed the analytical requirements for clinical diagnosis, where plasma metanephrine concentrations in healthy individuals typically range from 0.3–2.5 nmol/L [2]. The excellent linearity (R^2^ > 0.999), robust accuracy, and precision across validated ranges confirmed the suitability of this method for quantitative analysis and aligned with the validation criteria established in previous LC–MS/MS metanephrine assays [27,28]. 

In this study, a weak cation exchange approach was employed for SPE, allowing metanephrines to be retained on the SPE cartridge and eluted in a reduced volume relative to the original plasma sample. This concentration step enhances the detectability of analytes using LC-MS/MS, offering superior sensitivity and selectivity compared with traditional methods, such as electrochemical or fluorometric detection. The concentration step in SPE improves analyte enrichment, addressing the key analytical challenges in plasma metanephrine measurements [29]. 

To evaluate the matrix effect, the process efficiency was assessed by comparing the analyte response in the plasma spiked before extraction (Sample A) with that in the neat solution (Sample B). At the lowest concentration, metanephrine showed a process efficiency of 121%, exceeding the acceptable range of 100 ± 15%. This may be attributed to matrix-induced ion enhancement, which tends to be more prominent at lower analyte concentrations [30]. In addition, the variability in SPE recovery at low concentrations may have contributed to the elevated response. Because Sample A underwent SPE with the analyte present in the plasma, whereas Sample B lacked matrix components, differences in ionization efficiency or recovery could explain this discrepancy. 

Previous studies have reported similar matrix effects in plasma metanephrine analysis. The magnitude of matrix effects can vary depending on the sample preparation method, with SPE typically providing better matrix cleanup than simple protein precipitation [31]. However, completely eliminating matrix effects remains challenging, particularly at low analyte concentrations, where the signal-to-noise ratio is most susceptible to matrix interference [18,32].

The clinical significance of our findings must be interpreted in the broader context of the diagnostic performance. Although elevated process efficiency at low concentrations represents a deviation from ideal analytical conditions, this method nonetheless maintained acceptable accuracy and precision across all tested concentrations. These results suggest that the matrix effect, although present, did not compromise the quantitative reliability of the assay within a clinically relevant concentration range.

Several limitations should be acknowledged when interpreting our results. First, the elevated process efficiency observed at low metanephrine concentrations represented a deviation from the ideal analytical conditions and may have introduced systematic bias in the measurements near the LLOQ. Although the clinical impact appears minimal because accuracy and precision were maintained, this finding suggests that the method can be further optimized.

Next, matrix-effect evaluation was only performed using pooled plasma, an approach that masks the biological heterogeneity of clinical specimens. Therefore, it may underestimate ion suppression or enhancement arising from patient-specific factors, such as co-medications [33]. Individual samples may exhibit variable matrix interference. Additionally, certain drugs, including tricyclic antidepressants, sympathomimetics, and proton pump inhibitors, alter endogenous metanephrine concentrations or confound their measurements. Future validation studies should extend matrix-effect and interference testing to a broader range of patient samples representing diverse pharmacological and pathological backgrounds to strengthen the analytical robustness and clinical applicability of the method. 

Finally, this study did not include a clinical performance evaluation comparing our method with established diagnostic criteria or alternative analytical approaches. Although our analytical validation confirmed the technical adequacy of this method, a direct correlation with diagnostic outcomes would provide stronger evidence of its clinical utility.

Despite these limitations, the method showed acceptable process efficiency at all tested concentrations (86–121% across 0.6–5.0 nmol L^−1^). The accuracy and precision criteria were consistently met across the calibration range, thereby supporting the overall robustness and reliability of this method. Even with small deviations, further optimization, such as using matrix-matched calibration standards or improving the extraction protocol, may help reduce variability and increase the reproducibility. 

## 4. Materials and Methods

### 4.1. Chemicals and Reagents

High-performance liquid chromatography (HPLC)-grade water, acetonitrile, and methanol (J.T. Baker, Philipsburg, NJ, USA) were used for all experimental processes and LC solutions. LC-MS-grade formic acid (Optima, Waltham, MA, USA) and ammonium formate (Supelco, Bellefonte, PA, USA) were used as additives in the mobile phase. D,L-metanephrine hydrochloride (≥98%, HPLC grade, Sigma-Aldrich, St. Louis, MO, USA) and D,L-normetanephrine hydrochloride (≥98%, Sigma-Aldrich) were used in this study. Plasma calibration standards, controls, IS, and the tuning mix were obtained from ChromSystems (Munich, Germany). Plasma calibration standards and controls were rehydrated with HPLC-grade water, according to the manufacturer’s instructions.

### 4.2. Sample Preparation

The plasma calibration and control samples were stored at 4 °C. After rehydration and aliquoting, calibration and control samples were stored at −40 °C until use. The ISs were also stored at −40 °C. Patient plasma was collected from EDTA whole-blood samples, centrifuged at 2300× *g* for 10 min, and stored at 4 °C until use. 

### 4.3. SPE

Metanephrines were extracted using weak-cation exchange SPE. Strata-X solid-phase extraction chromatography (Strata-X-CW 33 µm Polymeric Weak Cation, 8E-S035-AGB) (Phenomenex, Torrance, CA, USA) was used. Cartridges were conditioned with methanol (1 mL) and equilibrated with 1mL of water. Plasma samples were prepared for loading, diluted with 0.1% formic acid in water, and centrifuged at 15,000× *g* for 10 min. Next, 1 mL of the diluted sample was added. During the washing step, 1 mL of methanol and 1 mL of water were added sequentially. Finally, 100 μL of 5% formic acid in acetonitrile was used for the elution. The eluted samples were dried under a positive-pressure system using nitrogen gas at 40 °C, 30 L/min for 1 h, and then reconstituted in a solution matching the initial LC conditions (Solvent A:Solvent B = 1:9).

### 4.4. Analytical Procedure

The analytical instrument consisted of a Sciex Exion liquid chromatograph combined with a Sciex QTRAP 5500 mass spectrometer (AB SCIEX, Framingham, MA, USA). The analytical column used in this experiment was an Acquity UPLC BEH Amide column (1.7 μm, 2.1 × 100 mm, P/N 186004801; Waters, Milford, MA, USA). The Hydrophilic Interaction Liquid Chromatography (HILIC) phase, coupled with ultra-HPLC, allows for the efficient separation and detection of metanephrines. The temperature of the column oven was maintained at 60 °C.

The mobile phases were:

- Solution A: 20 mM ammonium formate in 0.1% formic acid
- Solution B: 100% methanol.

The total flow rate was 0.5 mL/min. The equilibration and initial conditions for LC were set at 90% solution B. During the analysis, the concentration of solution B was maintained at 90% for 1 min, followed by a linear gradient from 90% to 65% for 1.5 min. It decreased to 40% after 1 min. The concentration of solution B was returned to 90% for equilibration and stabilization before the next run was performed. The run time was 5 min.

For MS analysis, electrospray ionization was used as the ion source, and collision-induced dissociation occurred in a Q2 collision cell. Positive ion mode was used exclusively to analyze metanephrines. The multiple reaction monitoring method was used to analyze known analytes because of its ability to evaluate multiple ions in a single run. The multiple reaction monitoring conditions, including the ion transitions of the precursor and product ions, retention time, declustering potential, collision energy, and collision cell exit potential, are listed in Table 6. The parameters for the mass spectrometer were as follows: curtain gas, 45.0 psi; collision gas, medium; ion spray voltage, 2500.0 V; temperature, 600.0 °C; ion source gas 1, 50.0 psi, gas 2, 55.0 psi; entrance potential, 10.0 V. MultiQuant MD 3.0.2 (AB SCIEX) was used to quantify metanephrines.

### 4.5. Method Selection and Optimization (Fit-for-Purpose) 

#### 4.5.1. SPE Sorbent Rationale 

We evaluated protein precipitation and polymeric reversed-phase SPE against weak cation-exchange (WCX) chromatography. We retained WCX because it reduced the matrix background and delivered consistent recoveries for protonated amines under acidic load and wash steps, and it yielded sharp peaks after acidified organic elution. 

#### 4.5.2. Chromatographic Mode and Additives 

Reversed-phase columns, including C18, provide limited retention for polar analytes. The amide HILIC phase provided stable retention and baseline separation of metanephrine and normetanephrine using volatile buffers (ammonium formate with formic acid) and high-organic initial conditions. We set the column temperature to an elevated hardware-safe level to minimize tailing.

#### 4.5.3. Mobile-Phase Composition 

We tuned the buffer ionic strength and pH (with formic acid) to balance the electrospray efficiency and chromatographic retention. The final composition maximized the signal-to-noise ratio (S/N) at the lower limit of quantification (LLOQ) and preserved peak symmetry and retention time (RT) stability within ±0.10 min.

#### 4.5.4. MRM Transitions 

Product-ion scans identified high-abundance fragment ions of metanephrine and normetanephrine. We optimized the collision energies and source parameters by direct infusion to maximize the sensitivity and minimize the in-source fragmentation. The highest-abundance transitions were assigned as quantifiers. We explored the qualifiers but did not implement them because the sensitivity at the LLOQ was insufficient. Identity acceptance was based on the quantifier transition, retention time agreement within ±0.10 min, and appropriate internal-standard response.

### 4.6. Method Validation

The laboratory-developed test followed the Bioanalytical Method Validation Guidance for Industry (FDA, Docket number FAD-2013-D-1020, 2018) and the Clinical and Laboratory Standards Institute guideline C62-A. The validation methods were partially modified [34,35].

Control samples (mass check-free metanephrine plasma controls, ChromSystems) were used to assess accuracy. Plasma controls at three concentrations (0.30, 0.93, and 4.94 nmol/L) were analyzed (n = 20) to determine accuracy (% bias). The average values and biases were calculated to determine the accuracy. The passing criterion was a bias of less than 15%, with the lowest level of bias being less than 20%.

Low- and high-level control samples were also used. To evaluate within-run precision, low (0.30 nmol/L) and high (4.94 nmol/L) concentration controls were measured for five days. To examine the between-run precision, two levels of control samples were measured in the morning and afternoon (time interval of >4 h) for 5 days. The accuracy of both evaluations was within 100 ± 15%. The CV was set to <20%.

Calibration standards (6PLUS1® Multilevel Plasma Calibrator Set Free Metanephrines, ChromSystems, Munich, Germany) were used to evaluate the linearity. The six calibration standards were tested five times. The average of five results was calculated and compared with the target value according to the standard concentration in a datasheet. The coefficient of determination (R^2^) of the regression exceeded 0.95, and the recovery was within 100 ± 15%.

Low and high concentrations of control samples were used to evaluate carryover (residual analyte detected in subsequent blank or low-concentration samples). Carryover was examined by consecutively measuring three high-concentration control samples and three low-concentration controls, repeated 10 times. To evaluate carryover, ten results of the first (low 1) and last (low 3) low-concentration measurements were statistically analyzed. F-tests were conducted for both groups (low 1 and 3). If the variances were equal, an equal-variance *t*-test was performed. A *p*-value < 0.05 indicated no significant difference between the two groups or no carryover effect. 

The lowest concentration in the control group was used to determine the LLOQ. The expected concentrations were determined by performing serial 1:2 dilutions of the manufacturer’s lowest calibrator. The expected concentrations were 0.307–0.061 nmol/L for metanephrines and 0.541–0.108 nmol/L for normetanephrine. Each diluted sample was analyzed five times. The average of the five results was statistically analyzed, and the accuracy was calculated. The accuracy should be within 100 ± 15%, with a CV% of less than 20%. The lowest concentration that satisfied these two conditions was considered the LLOQ for the analyte.

The matrix effects of the metanephrine detection methods, including SPE, were evaluated by comparing drug spiking before and after the sample preparation. Concentrations of 0.6, 1.3, 2.5 nmol/L for metanephrine and 1.3, 2.5, 5.0 nmol/L for normetanephrine were prepared. Three sample types were prepared as follows:

- Sample A: spiked with the drug and IS before SPE (plasma + analyte/IS (pre-[SPE])
- Sample B: drug- and IS-spiked reconstitution solution without SPE (90% acetonitrile + analyte/IS [no matrix])
- Sample C: spiked with the drug and IS after SPE before drying with nitrogen gas (plasma [post-SPE] spiked after extraction)

The results were calculated based on peak areas. The matrix factor was evaluated by comparing Samples B and C, with the difference being whether the matrix was plasma. Recovery was evaluated by comparing the differences in spiking timing. Spiking of the drug and IS before (Sample A) and after (Sample C) SPE allowed recovery from the SPE procedure. The process efficiency was evaluated by comparing samples A and B. ISs were used to normalize the peak area. The normalized results were calculated using the same procedure employed for routine clinical specimen reporting. The results were within the range of 100 ± 15%. 

For interlaboratory validation, 41 identical clinical samples were analyzed independently by our laboratory and Green Cross Corporation (Yongin, Republic of Korea). A comparative analysis of the two datasets was conducted using Passing–Bablok regression to evaluate systematic and proportional differences. 

## 5. Conclusions

We established and validated a sensitive and reliable LC-MS/MS method for quantifying metanephrine and normetanephrine levels in human plasma. The method met all major bioanalytical validation criteria and was suitable for routine clinical use to screen for catecholamine-secreting tumors. Although minor variability was observed at low concentrations, it did not compromise overall performance. This method offers significant advantages over traditional detection methods in terms of speed, accuracy, and diagnostic applicability.

## Figures and Tables

**Table 1:** Accuracy of plasma metanephrine and normetanephrine determined by SPE-LC-MS/MS.

Analyte	Nominal Concentration (nmol/L)	Mean	SD	Accuracy (%)
Metanephrine	0.30	0.30	0.01	101.5
	0.93	0.90	0.02	96.5
	4.94	4.76	0.06	96.3
Normetanephrine	0.67	0.65	0.02	98.1
	1.50	1.44	0.06	96.2
	8.26	7.91	0.20	95.7

SD, standard deviation.

**Table 2:** Within-run and between-run precision of plasma metanephrine and normetanephrine measurements.

Analyte	Nominal Concentration (nmol/L)	Within Run (n = 20)				Between Run (n = 20)			
		Mean	CV (%)	SD	Accuracy (%)	Mean	CV (%)	SD	Accuracy (%)
Metanephrine	0.30	0.30	1.4	0.00	99.8	0.30	1.7	0.01	100.7
	4.94	4.77	1.4	0.07	96.5	4.77	1.5	0.07	96.5
Normetanephrine	0.67	0.66	4.2	0.03	98.0	0.62	7.0	0.04	93.1
	8.26	8.13	1.8	0.15	98.4	7.95	3.0	0.24	96.3

CV, coefficient of variation; SD, standard deviation.

**Table 3:** Results of the carryover test for metanephrine and normetanephrine.

Analyte	Low 1 (n = 10)		Low 3 (n = 10)		F Test *p*-Value	t-Test *p*-Value
	Mean	SD	Mean	SD		
Metanephrine	0.31	0.03	0.30	0.03	0.34	0.29
Normetanephrine	0.69	0.13	0.62	0.04	0.45	0.07

SD, standard deviation.

**Table 4:** Lower limit of quantification (LLOQ) for plasma metanephrine and normetanephrine determined by SPE–LC-MS/MS.

Analyte	Expected Concentration (nmol/L)	Average Concentration (nmol/L, n = 5)	CV (%)	Accuracy (%)
Metanephrine	0.307	0.301	3.9	98.1
	0.245	0.247	2.8	100.7
	0.184	0.185	2.9	100.4
	0.123	0.126	5.5	102.8
	0.061	0.072	7.3	118.1
Normetanephrine	0.541	0.502	10.9	92.8
	0.432	0.409	11.0	94.6
	0.324	0.272	6.4	84.0
	0.216	0.150	37.1	69.6
	0.108	0.072	27.1	66.8

CV, coefficient of variation.

**Table 5:** Matrix effect, recovery, and process efficiency for metanephrine and normetanephrine (samples A–C).

Analyte	Analyte Concentration (nmol/L)	Recovery	Matrix Factor	Process Efficiency	IS-Normalized Recovery	IS-Normalized Matrix Factor	IS-Normalized Process Efficiency
		A/C (%)	C/B (%)	A/B (%)	(A_analytes_/C_analytes_)/ (A_IS_/C_IS_) (%)	(C_analytes_/B_analytes_)/ (C_IS_/B_IS_) (%)	(A_analytes_/B_analytes_)/ (A_IS_/B_IS_) (%)
Metanephrine	0.6	122%	94%	11%	108%	112%	121%
	1.3	122%	88%	12%	107%	102%	110%
	2.5	116%	97%	11%	111%	101%	112%
Normetanephrine	1.3	170%	58%	2%	86%	106%	92%
	2.5	140%	91%	3%	101%	111%	111%
	5.0	129%	89%	4%	112%	98%	110%

Sample A was pretreated with plasma samples spiked with analytes and IS. Sample B consisted of 90% ACN spiked with analytes and IS. Sample C was spiked with analytes, and IS was pretreated with analyte-free plasma. ACN, acetonitrile; IS, internal standard.

**Table 6:** Metanephrine parameters for multiple reaction monitoring.

Analyte	Precursor Ion (*m*/*z*)	Product Ion (*m*/*z*)	RT (min)	DP (V)	CE (V)	CXP (V)
Metanephrine	180.036	148.2	2.16	136	23	10
Metanephrine-d_3_	183.051	151.2	2.16	141	23	8
Normetanephrine	166.100	134.1	2.68	100	26	7
Normetanephrine-d_3_	169.400	137.2	2.68	100	26	7

RT, retention time; DP, declustering potential; CE, collision energy. CXP: collision cell exit potential.

## Data Availability

Raw LC–MS/MS data files (.wiff and .wiff.scan), processed quantitation tables (.csv), and the full validation reports/worksheets (accuracy, precision, selectivity, matrix effect, and carryover) are available from the corresponding author upon reasonable requests. Data is contained within the article.

## Abbreviations

The following abbreviations are used in this manuscript.

CV	Coefficient of variation
DOPA	Dihydroxyphenylalanine
HPLC	High-performance liquid chromatography
IS	Internal standard
LC-MS/MS	Liquid chromatography and tandem mass spectrometry
LLOQ	Lower limit of quantification
SPE	Solid-phase extraction
